# An experimental evaluation of the incidence of fitness-function/search-algorithm combinations on the classification performance of myoelectric control systems with iPCA tuning

**DOI:** 10.1186/1475-925X-12-133

**Published:** 2013-12-27

**Authors:** Guillermo A Camacho, Carlos H Llanos, Pedro A Berger, Cristiano Jacques Miosso, Adson F Rocha

**Affiliations:** 1 Faculty of Engineering, University of La Salle, Bogotá, Colombia; 2 Mechanical Engineering Department, University of Brasília, Brasília, Brazil; 3 Computer Science Department, University of Brasília, Brasília, Brazil; 4 Faculty of Gama, University of Brasília, Brasília, Brazil

**Keywords:** Artificial bee colony (ABC), Electromyography, Individual principal component analysis (iPCA), Myoelectric control, Particle Swarm optimization (PSO), Prosthesis, Pattern recognition

## Abstract

**Background:**

The information of electromyographic signals can be used by Myoelectric Control Systems (MCSs) to actuate prostheses. These devices allow the performing of movements that cannot be carried out by persons with amputated limbs. The state of the art in the development of MCSs is based on the use of individual principal component analysis (iPCA) as a stage of pre-processing of the classifiers. The iPCA pre-processing implies an optimization stage which has not yet been deeply explored.

**Methods:**

The present study considers two factors in the iPCA stage: namely *A* (the fitness function), and *B* (the search algorithm). The *A* factor comprises two levels, namely *A*_1_ (the classification error) and *A*_2_ (the correlation factor). Otherwise, the *B* factor has four levels, specifically *B*_1_ (the Sequential Forward Selection, SFS), *B*_2_ (the Sequential Floating Forward Selection, SFFS), *B*_3_ (Artificial Bee Colony, ABC), and *B*_4_ (Particle Swarm Optimization, PSO). This work evaluates the incidence of each one of the eight possible combinations between *A* and *B* factors over the classification error of the MCS.

**Results:**

A two factor ANOVA was performed on the computed classification errors and determined that: (1) the interactive effects over the classification error are not significative (*F*_0.01,3,72_ = 4.0659 > *f*_*AB*_ = 0.09), (2) the levels of factor *A* have significative effects on the classification error (*F*_0.02,1,72_ = 5.0162 < *f*_*A*_ = 6.56), and (3) the levels of factor *B* over the classification error are not significative (*F*_0.01,3,72_ = 4.0659 > *f*_*B*_ = 0.08).

**Conclusions:**

Considering the classification performance we found a superiority of using the factor *A*_2_ in combination with any of the levels of factor *B*. With respect to the time performance the analysis suggests that the PSO algorithm is at least 14 percent better than its best competitor. The latter behavior has been observed for a particular configuration set of parameters in the search algorithms. Future works will investigate the effect of these parameters in the classification performance, such as length of the reduced size vector, number of particles and bees used during optimal search, the cognitive parameters in the PSO algorithm as well as the limit of cycles to improve a solution in the ABC algorithm.

## Introduction

The loss of capabilities to manipulate objects with the hands is considered a high impact disease due to the physical, psychological and financial sequels related [1,2]. A current technological aid for upper limb amputation and deficiency is represented by the use of a myoelectric hand prosthesis. This device overcomes the inability to grasp and manipulate objects [3] and in some cases to sense and explore the surrounding world [4]. A myoelectric prosthesis is composed of two main systems: (a) the prosthesis and (b) the Myoelectric Control System (MCS), which is the topic of this work. The electromyographic signals (EMG) are used as an input of the MCS, which classifies patterns of the EMG signals and operate actuators in the prosthesis.

The conventional MCS classifies the myoelectric patterns in movement classes. The processing in the MCS is composed of three stages: feature extraction, dimensionality reduction and classification. Recently, Hargrove et al. [5] proposed a complementary architecture which improves the performance of the conventional MCS. This new architecture mixes 3 components: (a) a high crosstalk level EMG acquisition system, (b) an individual Principal Component Analysis (*iPCA*) transformation stage and (c) the conventional myoelectric control system. The main disadvantage of the iPCA projection is the dimensionality increment of the electromyographic patterns, in which the pattern dimension *N* is incremented by a factor corresponding to the control system’s number of classes *C*.

To deal with the dimensionality increment it is possible to compute a reduced iPCA transformation that generates just the *N*_1_ most discriminative dimensions instead of the complete set *C* × *N* (with *N*_1_ < *C* × *N*). The reduced iPCA transformation is used in [5] and requires an optimization process that intends to find a subset of *N*_1_ elements from a given set of *C* × *N* channels, warranting the maximum discrimination information in the selected subset. The optimization is based on a search strategy and a fitness function, i.e. a function that measures the quantity of discriminative information in the selected subset of channels.

The optimization in myoelectric control systems with iPCA tuning is explored by the authors in [6], where the analysis is focused on the effects of factors *A* and *B* over the performance of myoelectric control systems with iPCA tuning; with *A* representing the fitness function factor with two levels (*A*_1_ classification error and *A*_2_ correlation factor) and *B* representing the search algorithm factor with three levels (*B*_1_ sequential forward selection (SFS), *B*_2_ sequential floating forward selection, *B*_3_ artificial bee colony (ABC)). Our paper presents new results that complete the investigation in [6]. Specifically, we evaluate a new level for the *B* factor: *B*_4_ particle swarm optimization (PSO) for a total of eight treatment alternatives.

The paper is organized as follows: Section 'Related works’ discusses previous works in this research area. Section 'Background’ describes the principal component analysis, the problem of channel optimization, the search algorithms and the fitness functions considered. In Section 'Methods’ we present the experimental methodology. In Section 'Results’ the simulation results are given. In Section 'Discussion’ the results are discussed, and finally the conclusions are presented in Section 'Conclusions’.

## Related works

Different approaches for the control of myoelectric prostheses have been used. These can be grouped in two trends [7]: (a) coordinated control [8] and (b) sequential control which is the main topic of the current study. In the latter, the degrees of freedom are actuated one at a time, in a sequence, as one carries out a multifunction task. These systems are based on pattern recognition architectures where the patterns are represented by the EMG signal characteristics (*x*) and the classes are represented by the movements of the prosthesis (*y*).

Starting from one of the first works that made use of a multilayer perceptron (MLP) neural networks (NNs) [9-11], various classifiers such as linear discriminant analysis (LDA) [12-14], (neuro) fuzzy [15-20], Gaussian mixture models (GMMs) [21,22], hidden Markov models (HMMs) [18], and support vector machines (SVMs) [23-25] have been used. Some commonly investigated feature sets include time domain (TD) features: MAV, MAVS, ZC, SSC, WL [12,26], autoregressive (AR) coefficients [27], cepstral coefficients [28], the short-time Fourier transform (STFT), the wavelet transform (WT), the wavelet packet transform [13] (WPT), and concatenated TD and AR (TDAR) [21,29] features. These solutions have reached different classification errors, depending on the number of classes and other factors (see Table 1 for a summary of the different errors reported in last years). For instance, in [29] an error of 7.4% is reported in a problem with 7 movement classes and 8 electromyography channels. Recently, a new strategy has been proposed in which the temporal-spatial information, contained within muscle crosstalk, may implicitly add class discriminatory information to the classification problem [21]. This proposal has been investigated by Hargrove [5] observing a significative reduction in the classification error. In a problem with 7 movement classes and 6 EMG channels the system yielded a classification error of 1.9%.

**Table 1:** Summary of the classification parameters and errors reached in the last years

**Classifier**	**Channels**	**Classes**	**Features**	**Subjects**	**Error**	**Additional processing**	**Reference**	**Year**
MLP	2	4	MAV, MAVS, ZC, SSC, WL.	9	9.25%		[9]	1993
LDA, MLP	5	4	WPT. Transient	16	6.25%		[30]	1998
PCA, LDA	4	6	WPT. Stationary	11	6.80%		[13]	2001
LDA	4	4	TD. Stationary	12	5%	*Majority vote*	[12]	2003
GMM	4	6	AR6+RMS+TD. Stationary	12	3.10%	*Majority vote*	[21]	2005
						Suppress interclass data		
LDA	8	7	AR4+RMS. Stationary	30	7.40%	*Majority vote*	[29]	2007
						Suppress interclass data		
SVM	4	5	RMS +AR6. Stationary	11	3%	*Majority vote*	[23]	2008
						Suppress interclass data		
LDA	10	11	AR6 Stationary	10	6.50%		[5]	2009
LDA	10	11	AR6 Stationary	10	5%	iPCA Transformation	[5]	2009

**Figure 1:**
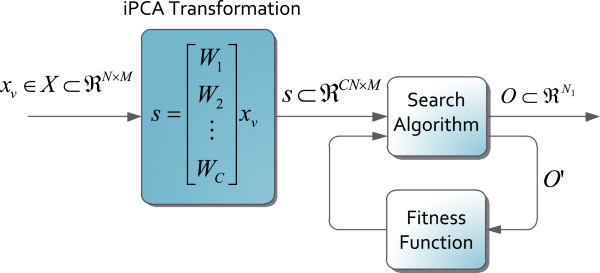
**Optimization scheme used in the parameter configuration stage.** *W*_*i*_ is a PCA matrix transformation of size *N* × *N* for *i* = 1,…,*C*. Note that each *W*_*c*_ (for *c* = 1,2,…*C*) matrix has a *N* × *N* size, then the projected vector *s* has an incremental size of *CN* × *M*.

The proposal in [5] mixes 3 components: (a) a high crosstalk level EMG acquisition system (i.e. an acquisition system with an special distribution of electrodes that permits that the signal generated by the *M*_1_ muscle can be detected by the electrode associated with the *M*_2_ muscle, where muscles *M*_1_ and *M*_2_ are near [7]), (b) an iPCA transformation stage and (c) the conventional myoelectric control system. The main disadvantage of the iPCA projection is the dimensionality increment of the electromyographic patterns, in which the original pattern dimension *N* is incremented by a factor corresponding to the control system’s number of classes *C*, i.e. it generates a new pattern with dimension *C* times *N*. To deal with the dimensionality increment it is possible to compute a reduced iPCA transformation that computes just the *N*_1_ most discriminative dimensions instead of the complete set *C* × *N* (with *N*_1_ < *C* × *N*). This solution requires an optimization search to find a subset of *N*_1_ elements from a given set of *C* × *N* channels, warranting the maximum discrimination information in the selected subset. Because of the time of response of these systems, the optimization process must be conducted during a configuration stage, before the classification tasks. This optimization uses a search algorithm and a fitness function as depicted in Figure 1. The results in [5] were computed using a combination of SFS (*sequential forward selection*) algorithm and the *classification error* fitness function. Despite important reported advantages, there are also some disadvantages for that configuration: (1) The SFS algorithm presents the *nesting effect*, i.e., once a channel has been selected there is no possibility of discarding it [31]. (2) The SFS algorithm does not use random components [32]; note that this type of components can help by finding different solutions and potentially lead to better ones [33] in problems with more than one local optimum. (3) The use of the classification error as the fitness function requires a supervised procedure, in which the classes associated to each pattern are known, being computationally expensive, due to the need for evaluating the classifier’s output at each iteration.

In order to obtain a solution to the previous described disadvantages the authors propose and evaluate in [6], a set of novel configurations for the iPCA tuning, based on two factors: (*A*) fitness function and (*B*) search algorithm. The *A* factor with two levels: (*A*_1_) classification error and (*A*_2_) correlation factor. The *B* factor with three levels: (*B*_1_) sequential forward selection (SFS), (*B*_2_) sequential floating forward selection and (*B*_3_) artificial bee colony (ABC). In this context, this article evaluates a new level for the *B* factor: *B*_4_ particle swarm optimization (PSO). The overall results suggest an advantage on the use of the PSO algorithm with respect to other studied algorithms, with regard to the running time during the training stage.

## Background

### Principal Components Analysis

PCA is an orthogonal linear transformation used to convert a set of observations of possibly correlated variables into a set of values of uncorrelated variables called principal components. Given *M* observations of an *N* dimensional random vector *z*, the PCA transformation is performed by firstly subtracting the mean of the vector from *z*[5], *x* = *z* - *E*{*z*}, computing the *N* × *N* covariance matrix *C*_*x*_ = *E*{*xx*^*T*^} and, then, applying *s* = *Wx*, where *s* is the vector of the main component and *W* is the matrix in which each column is an eigenvector of *C*_*x*_. Usually the *M* observations would typically be samples taken from any *C* possible classes. This is known as *universal* PCA (uPCA) or global PCA [34]. This property of ignoring class information allows us to argue that PCA is suboptimal for classification purposes [35].

A recent variation, called individual PCA (iPCA) [34,35], groups the *M* observations according to their class membership. Separate projection matrices *W*_1_,…,*W*_*C*_ with size *N* × *N* are found for each class (see Figure 1). This set of matrices can be interpreted as a unique *CN* × *N* size transformation matrix *W*_*iPCA*_ formed by the concatenation of rows of the separate projection matrices. The iPCA method effectively “tunes” the data prior to classification and has been shown to improve classification accuracies for some pattern recognition problems [36]. The main drawback of this method is the linear increment of the dimensionality of the patterns with the number of classes *C*. To overcome this problem, a reduced iPCA transformation matrix *W*_*R*_ is defined in [5]. This solution uses a transformation matrix with the best *N*_1_ basis of the *W*_*iPCA*_ matrix (*N*_1_ < *CN*).

### Channel optimization

The optimization task of finding a subset of *N*_1_ elements from a given set of *CN* channels can be interpreted as a discrete optimization problem (integer elements from the selected subset). The optimization scheme in Figure 1 is used to carry out this task. In this scheme, a validation data set *x*_*v*_ is projected with the *W*_*iPCA*_ matrix. The projected pattern *s* is a *CN* × *M* size vector. The vector *O*^′^ is a subset of the *CN* dimensions in *s* and its components change at each iteration, following a specific strategy defined by the *search algorithm*. The *fitness function* evaluates the quality of an EMG pattern with regard to the discriminative information. The evaluated patterns by the fitness function are established by the rows of *s* which compose the *O*^′^ vector. When the stop conditions are reached, the scheme provides the selected channel subset *O* as output.

### Search algorithms

The search algorithms treated in this study are the following: (a) *Sequential Forward Selection* (SFS), (b) *Sequential Floating Forward Selection* (SFFS), (c) *Particle Swarm Optimization* (PSO), and (d) *Artificial Bee Colony* (ABC); then, the same will be described.

### Sequential Forward Selection (SFS)

This is one of the first developed search methods in the literature related to feature selection issue. The search procedure consists of the following steps: (a) compute the fitness function for each of the *CN* channels and then select the channel with the best value, and (b) form all possible two dimensional vectors that contain the winner from the previous step and compute the fitness for each of them, (c) select the vector with the best value, (d) continue the process until the *N*_1_ vector length has been completed. The main drawback of this algorithm is the nesting effect; that is, once a channel has been selected there is no possibility to discard it [32].

### Sequential Floating Forward selection (SFFS)

This is a suboptimal search algorithm proposed by Pudil et al. in 1994 [31] with the purpose of eliminating the nesting effects of the SFS algorithm. This algorithm begins the search with an initial subset of two channels. For each subsequent iteration, two tasks are necessary: (a) search the candidate channel which minimizes the fitness function and add it to the selected subset *O*^′^, (b) verify if the fitness function can be optimized by replacing a channel from the selected subset *O*^′^. Consequently, the SFFS search is performed dynamically, incrementing and decrementing the selected channels in the subset *O*^′^ until reach the target length *N*_1_. An efficient way to implement this algorithm is presented in [31,37].

### Particle Swarm Optimization (PSO)

This is a population based stochastic optimization technique developed by Eberhart and Kennedy in 1995 [38]. In this algorithm, it is assumed that there exists a swarm with *S* particles. Consider that the search space is *N*_1_-dimensional, and the *i-th* particle of the swarm can be represented by a dimensional position vector *x*_*i*_ = (*x*_*i*1_,*x*_*i*2_,…,*x*_*i*_*N*_1_). The velocity of the particle is denoted by *v*_*i*_ = (*v*_*i*1_,*v*_*i*2_,…,*v*_*i*_*N*_1_). Additionally, consider that the best individual position for the *i* particle is $p_{{\text{best}}_{i}}=(p_{i1},p_{i2},\ldots ,p_{i}N_{1})$ and also that the first and second best position explored so far are *gfbest* and *gsbest* respectively. The velocity of the particle and its position are updated according to (1) and (2).

(1) $$\begin{array}{ll} \;v_{\text{ij}}(t+1) & =w*r(v_{\text{ij}}(t))+c_{1}*r_{1}*r(\text{pbes}t_{\text{ij}}-x_{\text{ij}}(t))+c_{2}*r_{2}*r(\text{gfbes}t_{\text{ij}}-x_{\text{ij}}(t))\\ & \;+c_{3}*r_{3}*r(\text{gsbes}t_{\text{ij}}-x_{\text{ij}}(t)) \end{array}$$

(2) $$\begin{array}{l} x_{\text{ij}}(t+1)=x_{\text{ij}}(t)+r(v_{\text{ij}}(t+1)) \end{array}$$

where, *i* is the particle index for *i* ∈ 1,…,*S*; *j* is a specific dimension for *j* ∈ 1,…,*N*_1_; *w* is the inertia weight; *r*_1_, *r*_2_ and *r*_3_ are random numbers uniformly distributed in the range (0,1); *c*_1_ is a cognitive parameter; *c*_2_ and *c*_3_ are social parameters; *r*(.) is a rounding function used to adapt the original PSO for the discrete optimization problem [39,40].

The purpose of including the second best particle *gsbest* in the velocity update equation is to increase the diversity and slow convergence by addressing each particle to move toward the weighted sum of both the best and second best solution the swarm has generated [41]. Considering a minimization problem the local best of each particle is updated according to (3).

(3) $${\text{pbest}}_{i}(t+1)=\left\{\begin{array}{ll} {\text{pbest}}_{i}(t) & \text{if}\;f({\text{pbest}}_{i}(t))\leq f(x_{i});\\ x_{i}(t) & \text{if}\;f({\text{pbest}}_{i}(t))>f(x_{i}); \end{array}\right)$$

where, *f*(.) is the fitness function. Finally, the first and second global best of the swarm are updated according to (4) and (5).

(4) $$\text{gfbest}(t+1)=\text{arg}{\text{min}}_{p\in {\text{pbest}}_{i}}\;f(p(t+1))$$

(5) $$\text{gsbest}(t+1)=\text{arg}{\text{min}}_{p\in {\text{pbest}}_{i}\setminus g\;\text{fbest}(t+1)}\;f(p(t+1))$$

It is possible that the solution computed with the PSO algorithm contains repeated elements (i. e. that the vector *O*^′^ has repeated elements). This leads to EMG patterns with high level of redundancy and it does not benefit the localization of the global minimum on the considered fitness functions. To overcome this, a modification on the conventional PSO algorithm was added. Whenever the search process leads to a solution with repeated elements, then, that solution will be penalized with a high fitness value and without evaluating the fitness function. This will hinder the EMG patterns with high level of redundancy from being selected as the optimal solutions in the minimization problem.

### Artificial Bee Colony (ABC)

This is an optimization technique proposed in 2005 by Karaboga [42]. In ABC, each solution to the optimization problem is called *food source* and is represented by a *N*_1_-dimensional vector. The colony of artificial bees contains three groups of bees: employed bees, onlookers and scouts. The first half of the colony consists of employed artificial bees and the second half are the onlookers. For each food source there is only one employed bee. Therefore, the number of employed bees is equal to the number of food sources. An employed bee whose food source has been exhausted becomes a scout. The ABC algorithm can be implemented in two steps [43]. 

• Initialization step: the algorithm generates a random initial solution set of length *S*. Each solution *x*_*i*_(*i* = 1,2…,*S*) is a *N*_1_-dimensional vector. The employed bees measures the nectar amount of each solution, return to the hive and share the nectar information with the onlooker bees.

• Iterative step: the solution set is submitted to repetitive search cycles. During these cycles, the bees change its memory contents, searching the food source with the best fitness. Each bee is able to remember just one food source localization *x*_*i*_. Each cycle is computed in three phases: (a) *employed phase*, at this phase each employed looks for a new food source *v*_*i*_ around its current position *x*_*i*_ using (6). If the nectar quantity of this new position improves the previous one, then the bee position is updated. Once each employed bee has finished this phase, a probability factor *p*_*i*_ is computed (7). Using this factor indicates the nectar level at each *x*_*i*_ food source. (b) *Onlooker phase*, at this phase the onlooker bees use the probability factor *p*_*i*_ of each employed bee and select a *x*_*i*_ food source. Afterwards, a new *v*_*i*_ food source around the selected neighborhood localization is computed (6). Finally, if the fitness value of this new position improves the previous one, then the bee position is updated. (c) *Scout phase*, whenever the exploration of a *x*_*i*_ food source does not finish with a solution improvement, a counter increments the number of trials of that food source. If the value in this counter is greater than a threshold *C*_*lim*_, this *x*_*i*_ food source is discarded and a new food source is randomly selected by a scout bee.

(6) $$v_{\text{ij}}=x_{\text{ij}}+\phi_{\text{ij}}(x_{\text{ij}}-x_{\text{kj}})$$

where *k* ∈ (1,2,…*S*) and *j* ∈ (1,2,…*N*_1_) are randomly chosen indexes, with *k* ≠ *i*. *ϕ*_*ij*_ is a random number in the range [-1,1] that controls the production of food source positions around *x*_*i*_.

(7) $$p_{i}=\frac{f(x_{i})}{\sum_{n=1}^{S}f(x_{n})}$$

where *f*(.) represents the fitness function value and *S* represents the number of food sources.

### Fitness functions

A fitness function is a particular type of function used to summarise, as a single figure of merit, how close a design solution is to achieving the setting aims. In this case, the aim is related with the maximum discrimination information in a subset of *C* × *N* channels obtained after the iPCA transformation. Two fitness functions have been used in this study: (a) *Classification error*, and (b) *Correlation factor*, which will be described here:

### Classification error

**Figure 2:**
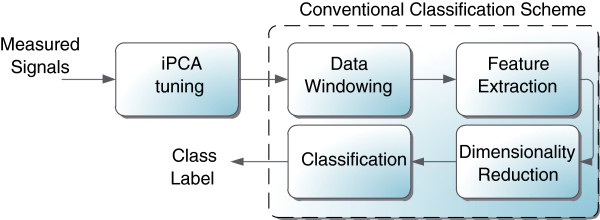
**Steps of the pattern recognition based myoelectric control system with iPCA tuning **[5]**.** The dotted line shown in the figure is the conventional classification architecture for myoelectric control systems. The measured signal is represented by a generic vector *x* and the class label by a generic vector *y*.

This is the same factor that has been used to measure the performance of the myoelectric control system; therefore, it is the ideal function to compare the discriminative information of the EMG patterns. This factor is computed as the relation between number of incorrect decisions and the total number of decisions of the system. To compute this relation, is necessary to know the predicted class vector $\overset{ˆ}{y}$ and, therefore, the classification scheme composed of the block diagrams inside the dotted line in Figure 2 (i.e. the conventional myoelectric system) must be performed. Consequently, there is a significant computational complexity involved in classification error computation.

### Correlation factor

Several factors have been proposed to quantify the amount of common signals presented between the two dimensions of a signal, the most common being the cross correlation factor [44]. The equation used to compute this factor for a discrete two dimension vector *x* is

(8) $$C_{x}(x_{1},x_{2})=E[(x_{1}-\mu_{1})(x_{2}-\mu_{2})]$$

where *E* is mathematical expectation and *μ*_*i*_ = *E*[*x*_*i*_]. For *N*-dimensional vectors, the correlation coefficient matrix is more used, where (9) defines each element of the matrix; each element varies within the range [1,-1].

(9) $$R_{x}(p,q)=\frac{C_{x}(p,q)}{\sqrt{C_{x}(p,p)C_{x}(q,q)}}$$

for *p* = 1,…,*N*; *q* = 1,…,*N*.

*R*_*x*_ is an *N* × *N* matrix with correlation coefficients computed for each pair of dimensions of the vector *x*. This matrix is symmetric and has ones in its main diagonal; therefore, to quantify the correlation level it is enough to consider the lower or upper diagonal elements. An alternative considering the lower elements is

(10) $$f_{c}=\text{sum}(\text{tril}(\left|R_{x}\right|))$$

where tril(.) is a function that returns the lower triangular elements of the matrix without the main diagonal ones. To limit the correlation factor between [0,100] we have defined the expression in (11), which has been used for computing the correlation factor between dimensions of the EMG signal.

(11) $$F_{c}=100\times \frac{2f_{c}}{N^{2}-N}$$

### Myoelectric Control Systems with iPCA tuning

Figure 2 depicts the myoelectric control scheme with iPCA tuning [5], which uses an iPCA transformation for tuning input patterns. This transformation is a variation of the PCA (*Principal Component Analysis*) that was initially used for improving the classification performance in problems of face recognition [34]. The purpose of the iPCA transformation is to generate a new signal space where the discriminative information related to the movement class is amplified, as long as other types of information are attenuated. However, there is a drawback: the patterns’ dimensions increase by a factor of *C*. Considering an input pattern corresponding to *N* EMG channels and a classification system with *C* classes, one has to use of a transformation matrix *W*_*iPCA*_ with size *CN* × *N*. Therefore, the projection of the input signal results in a new dimension pattern *CN* (see Figure 1) [5].

A reduced iPCA transformation can be defined for reducing the effects of dimension pattern increments. In this solution, it is necessary to compute a reduced iPCA transformation matrix *W*_*R*_. This matrix projects the input patterns and generates just the *N*_1_ most discriminative dimensions at the output (with *N*_1_ < *CN*). To compute *W*_*R*_ it is necessary to solve the block diagram in Figure 1. The output of this scheme is the vector *O*, which contains *N*_1_ channels selected from the *CN* set. Using this vector, the relation between the iPCA transformation matrix and the reduced iPCA transformation matrix is defined, as stated in (12), where the Matlab [45] notation for sub-matrices is used.

(12) $$W_{R}=W_{\text{iPCA}}(O,:)$$

## Methods

**Figure 3:**
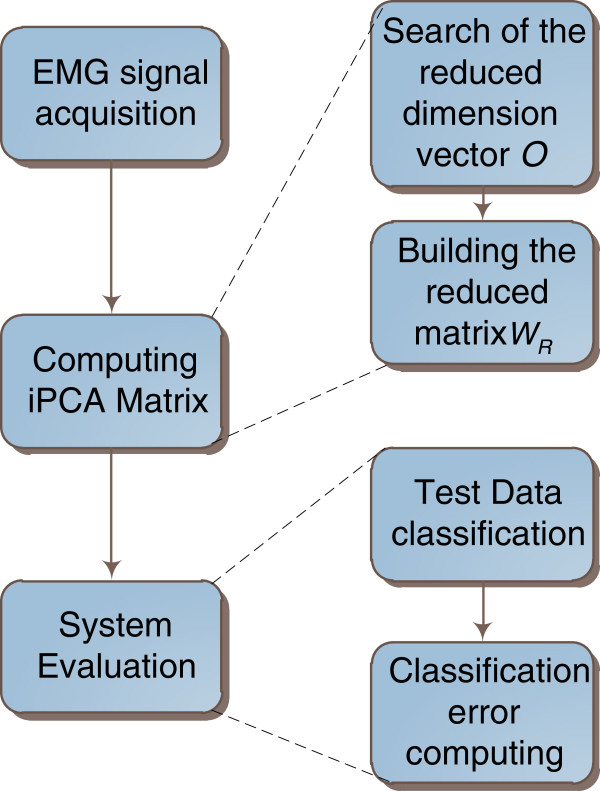
**Sequence used to evaluate the classification error.** Note that the blocks Computing iPCA Matrix and System Evaluation must run eight times, i.e. one iteration for each of the four search algorithms with the first fitness function (classification error) and one iteration for each of the four search algorithms with the second fitness function (correlation factor).

In order to evaluate the classification performance for each fitness-function/search-algorithm combination, a methodology based in three steps has been applied (namely, *EMG signal acquisition*, *computing the reduced iPCA matrix*, and *system evaluation*) which are depicted in Figure 3.

### EMG signal acquisition

We used data collected by the University of New Brunswick - Canada, with authorization of Dr. Levis Hargrove. These data were acquired in an experiment approved by the University of New Brunswick’s Research Ethics Board [5]. The data were sampled from ten healthy subjects performing eleven motion classes. EMG signals were acquired from ten sites on the forearm using adhesive duotrodes manufactured by 3M. These signals were amplified to guarantee potentials in range [+5,-5]*V* and a bandwidth of 1*Hz* to 500*Hz*. Afterwards, the signals were sampled at 1*KHz* and quantized with a 16-bit resolution.

Experimental data were collected during eight trials. Each trial consists of two repetitions of eleven motion classes performed in sequential order, namely: (1) *wrist pronation*, (2) *wrist supination*, (3) *wrist flexion*, (4) *wrist extension*, (5) *hand open*, (6) *key grip*, (7) *chuck grip*, (8) *power grip*, (9) *fine pinch grip*, (10) *tool grip*, and (11) *rest position* or no movement.

**Figure 4:**
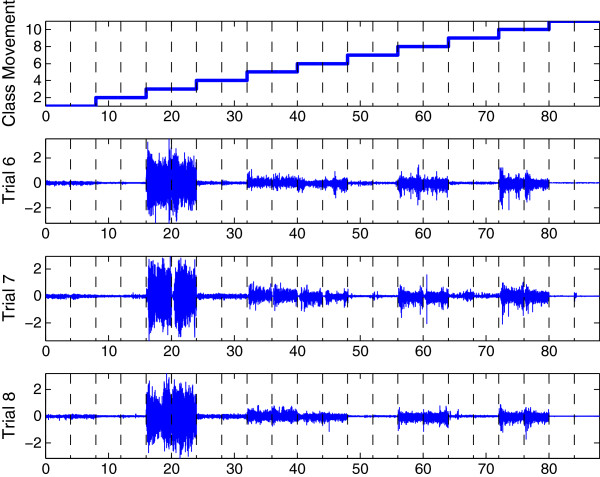
**Acquired signal with the sequence of movements from 1 to 11.** Signals illustrated correspond to channel 3 during trials 6 to 8 and user 1. Dotted lines indicate the held time for the contraction (4 s). Note that the movements were evaluated in sequential ascending order for all trials.

The intensity of the contraction was determined by the subject, but they were encouraged to contract to a level that they comfortably repeated throughout the experiment. During all trials, subjects elicited the contraction from the rest position, held the contraction for 4 s and then returned to the rest position for a predefined inter-motion class delay period. Trials 1, 2, 3 and 4 used intermotion class delay periods of 3, 2, 1 and 0 s respectively. Trials 5–8 used inter-motion class delay periods of 2 s. The result is a data set composed of myoelectric signals *x* in each channel and a vector of target classes associated *y*. A sample of the collected data excluding the intermotion class delays is depicted in Figure 4.

In the present study, the EMG data recorded from trials 1 and 3 were used as a training data set. EMG data recorded from trials 2 and 4 were used as a test data set. And finally, EMG data recorded from trials 5 and 6 were used as a validation set to resolve the optimization problem defined in the Background Section. Otherwise, for all these sets, the inter-motion class delays were excluded such as reported in [5].

### Computing the reduced iPCA matrix

**Figure 5:**
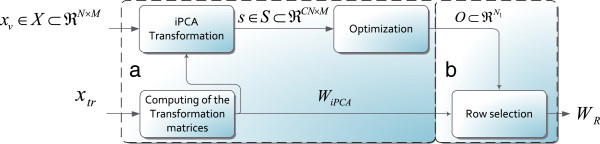
**Blocks for computing the reduced iPCA Transformation Matrix.** **(a)** search of the reduced dimension vector *O***(b)** building of the reduced iPCA transformation matrix *W*_*R*_. Note that this process is conducted during a configuration stage, before the classification.

This step has been implemented in two sequential tasks depicted in Figure 5: (a) search of the reduced dimension vector *O*, and (b) building of the reduced iPCA transformation matrix *W*_*R*_. The first task began with the computing of the iPCA transformation matrices using the training data *x*_*tr*_. Afterwards, the validation data *x*_*v*_ were projected with iPCA transformation matrices and the transformed patterns *s* were used to run the optimal subset channel search. The result of this search is the vector *O* composed of the *N*_1_ channels with most discriminative information. This search was evaluated with the eight possible combinations of fitness-function/search-algorithm. Therefore, at the end of this task the subsets *O*_*ij*_ were obtained, where the sub-indexes *i*, *j* indicate the fitness function and the search algorithm, respectively; for *i* = 1,2; *j* = 1,2,3,4.

In this case, for all search algorithms the *N*_1_ parameter was set to 30, such as recommended in [5]. This value provides good classification accuracy.

The SFS algorithm was configured with just one stop criterion, namely: length of the selected subset equal to *N*_1_.

The SFFS algorithm used two stop criteria: (1) the same defined in SFS and (2) the maximum number of iterations (*C*_*max*_ = 120). The latter was selected considering tests which show a mean number of iterations of 78.3 (with a standard deviation of 23.04) to find a solution. The PSO and ABC algorithms used other kind of parameters summed up in Table 2 and Table 3, respectively. For both fitness functions the optimization problem was defined as a minimization and the parameters were set with the next criteria:

**Table 2:** PSO algorithm parameters

**Symbol**	**Parameters**	**Value**
*ε*	Minimum value of fitness function	0
*c*_**1**_	Cognitive parameter	2
*c*_**2**_	Social parameter	2
*c*_**3**_	Social parameter	1
*w*	Inertial weight	[0.8, 0.1]
*S*	Swarm size	10
[*v*_*m* *i* *n*_,*v*_*m* *a* *x*_]	Velocity limits	[-109, 109]
[*x*_*m* *i* *n*_,*x*_*m* *a* *x*_]	Position limits	[1, 110]
*C*_*m* *a* *x*_	Maximum number of iterations	120

**Table 3:** ABC algorithm parameters

**Symbol**	**Parameter**	**Value**
*ε*	Minimum value of fitness function	0
*S*	Number of food sources	10
[*v*_*min*_,*v*_*max*_]	Limit values	[1, 110]
*C*_*max*_	Number of maximum cycles	120
*C*_*lim*_	Limit of cycles to improve a solution	6

For the PSO algorithm the minimum value of cost function (*ε*) was defined by the minimum possible value for the classification error and correlation factor (in this case equal to zero). The number of particles (*S*) was set to 10; for greater values, the search time was increased and the time for the experiments could exceed the available time (2 months to get experimental data), specially when the classification error were used as fitness function. For lower values, the search performance could be compromised due to the characteristics of population based stochastic optimization algorithms. The limit values (*v*_*min*_, *v*_*max*_, *x*_*min*_, *x*_*max*_) were defined by the dimension of the search space: *C* × *N*, where *C* is the number of movement classes (*C* = 10) and *N* is the number of acquisition channels (*N* = 11). The maximum number of iterations *C*_*max*_ was set to 120; this value showed good results for both fitness functions. The algorithm used similar values for cognitive and social parameters (*c*_1_, *c*_2_ and *c*_3_) allowing a neutral strategy, i.e. a strategy were the confidence in the experience of each particle is equal to the confidence in the experience of the swarm. The inertial weight *w* decreases linearly allowing that the particles polish the search in the last iterations; this configuration induces an effect of global search in the first iterations and local search at the last ones.

In the ABC algorithm the parameters were configured having two requirements in mind: (a) high performance in the optimal search, (b) assurance of conditions to make fair comparisons between bio-inspired algorithms. Then, the number of food sources (*S*) was configured to 10, equal than the number of particles in the PSO algorithm. The limit values (*v*_*min*_, *v*_*max*_) were defined by the dimension of the search space: *C* × *N*. The number of maximum cycles (*C*_*max*_) was set 120, equal that the maximum number of iterations in the PSO algorithm. The minimum value of the fitness function was set to 0, because this was the minimum value achievable by both fitness functions: classification error and correlation factor. The limit of cycles to improve a solution (*C*_*lim*_) was configured to 6; this value showed good classifications performances and affordable times of search.

To compute the classification error fitness function, the processing scheme formed by the blocks inside the dotted line in Figure 2, (i.e. the conventional myoelectric control system) has been used. The configuration was the following: overriding windowing feature extraction [12] with lengths of 150 ms and sliding windows of 25 ms, features conformed by the first 6 autoregressive coefficients (AR6) [18], dimensionality reduction method based on Uncorrelated LDA (ULDA) [29] and LDA (*Linear Discriminant Analysis*) classifier [46]. At this stage, each channel was independently used to train and, subsequently, to test the control system, such as presented in [5].

At the second task (row selection block in Figure 5) we used equation (12) to compute the reduced iPCA transformation matrix corresponding to each pair fitness function/search algorithm. Note that, at the output, eight reduced transformation matrices $W_{R}^{\text{ij}}$ were obtained as consequence of an optimization procedure computed eight times (the sub-indexes *i*, *j* indicate the fitness function and the search algorithm, respectively).

### System evaluation

**Figure 6:**
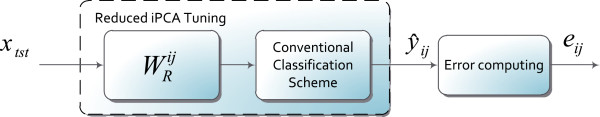
**Blocks for system evaluation.** The dotted line shown in the figure contains the test data classification tasks.

The last proceeding of the methodology was the evaluation of the classification system. This was carried out in two phases as depicted in Figure 6: (a) the test data (*x*_*tst*_) classification, and (b) the classification error computing. The first phase was achieved with the iPCA preprocessing and the conventional classification scheme (see Figure 2), using the same configuration described for the classification error fitness function computing. This process was computed for each of the reduced iPCA transformation matrices $W_{R}^{\text{ij}}$; the output of this phase was the predicted class vector ${\overset{ˆ}{y}}_{\text{ij}}$. The second phase compares the vector ${\overset{ˆ}{y}}_{\text{ij}}$ with the target vector *y* to compute the classification error as defined in equation (13). The classification error was individually evaluated for each user *k* of the system.

(13) $$e_{\text{ij}}(k)=100\times \frac{\text{sum}(y\neq {\overset{ˆ}{y}}_{\text{ij}}(k))}{\text{length}(y)}$$

## Results

The simulations proposed in this paper were performed on the Matlab platform and are based on the myoelectric control toolbox presented in [29]. Nevertheless, we have made some modifications and added some new functionalities to the myoelectric control toolbox presented in [29]. These modifications were necessary to appropriately parse the EMG data base that had a different structure than the used in [29]. The added functionalities were done for configuring and implementing all the iPCA related functions, namely: optimal search, transformation matrices computing projections and performance indicators computing. The resulting simulator is a powerful tool for experimentation with iPCA myoelectric control, designed on a modular fashion that permits evaluation of different approaches in each of the processing steps. Here we show results separated in two main stages: (*a*) first, the results registered during reduced iPCA transformation matrix computing, and secondly, (*b*) results registered during EMG pattern classification.

**Figure 7:**
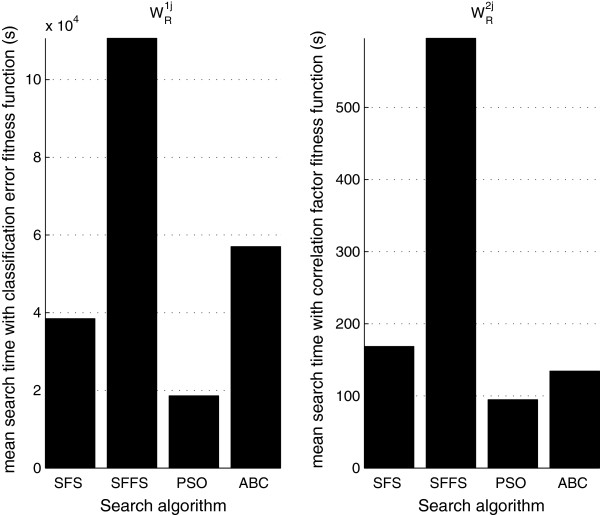
**Comparison of search time used for each of the eight treatment alternatives evaluated during optimization.** The search time was computed as the product between the number-of-iterations and the mean-iteration-running-time.

**Figure 8:**
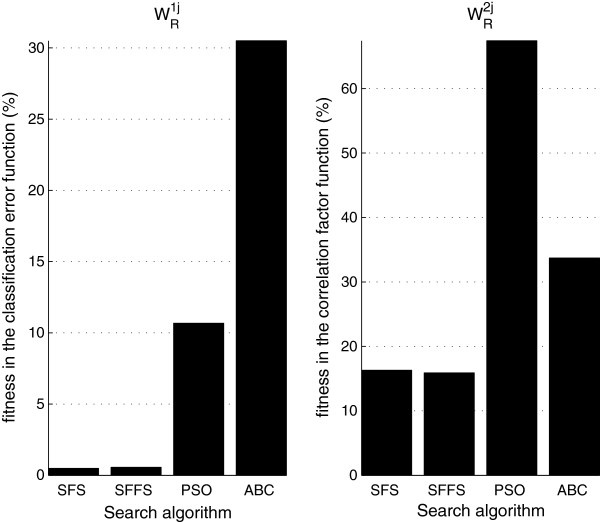
**Comparison of the fitness value reached with each of the eight treatment alternatives during the optimization search.** The sequence of algorithms in each figure was selected according to the descending order of fitness.

The results of the first stage of the system are shown in Figure 7 and Figure 8. Figure 7 depicts the mean search time, i.e. the time necessary to compute the optimal vector *O*. The left figure shows the search time for classification error fitness function and the right figure for correlation factor fitness function. Complementary information on this subject is presented in Table 4. This table summarizes the mean values of the number of iterations and the time of each single iteration for each fitness-function/search-algorithm combination. Note that both bio-inspired algorithms were stopped by the maximum iterations criteria (120 iterations); this means that the time performance comparison among bio-inspired algorithms just depends on the time of each single iteration of the search algorithm. The SFS algorithm was stopped when reached 30 iterations and the SFFS have different mean iterations number for each fitness function. The times in Table 4 have been computed on a PC with 2.8GHz processor, 8Gb RAM memory and 4 cores. Figure 8 depicts the mean fitness value reached during the optimal search. This value is presented for the eight evaluated combinations of search algorithm and the fitness function.

**Table 4:** Comparison of number-of-iterations/mean-iteration-running-time for the eight treatment alternatives during optimization

**Fitness function**	**SFS**	**SFFS**	**PSO**	**ABC**
*Class. error*	30/1282s	64.9/1705s	120/155s	120/475s
*Corr. factor*	30/5.62s	78.3/7.61s	120/0.79s	120/1.12s

**Figure 9:**
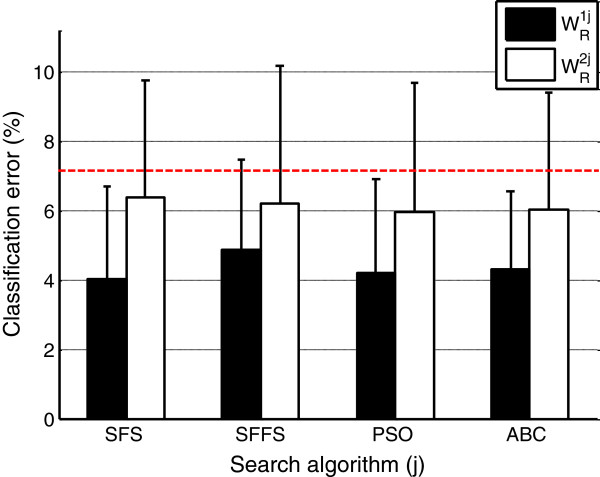
**Comparison of the classification error reached with each of the eight treatment alternatives.** The error bar represents one standard deviation of intersubject variability. The red line indicates the mean classifcation error obtained when the EMG patterns were classified without the iPCA transformation.

The results of the next main stage (the EMG pattern classification stage) are shown in Figure 9. This figure displays the mean classification errors generated when each of the eight reduced matrices $W_{R}^{\text{ij}}$ were used in the myoelectric control system. The black bars indicate the classification errors obtained with the transformation matrix $W_{R}^{1j}$ (i.e. the transformation matrices computed with the classification error fitness function) and white bars indicate the classification errors obtained with the transformation matrix $W_{R}^{2j}$ (i.e. transformation matrices computed with the correlation factor fitness function). The vertical line on the bars represents one standard deviation of inter-subject error variability. The red line indicates the mean classification error obtained when the EMG patterns were classified without the iPCA transformation.

A two factor analysis of variances (ANOVA) was performed over the error classification results, which has determined the following behaviors: (a) there was no statistical evidence of the dependence between the interaction of both factors (i.e. search algorithm and fitness function) and the classification error (*F*_0.01,3,72_ = 4.0659 > *f*_*AB*_ = 0.09), (b) there was statistical evidence of the dependence between the fitness function factor and the classification error (*F*_0.02,1,72_ = 5.0162 < *f*_*A*_ = 6.56), and (c) there was no statistical evidence showing the dependence between the search algorithm factor and the classification error factor, (*F*_0.01,3,72_ = 4.0659 > *f*_*B*_ = 0.08).

## Discussion

The analysis of simulation results may be performed taking into account two major aspects: (a) the computational complexity of the search process, and (b) the overall classification accuracy, using a fitness function. The metric used to measure the computational complexity was the number of iterations and the computational time of each iteration (see Table 4). The product of these two metrics defines the search time (i.e. the time necessary to find an optimal solution) and gives an estimate of the computational complexity. With regard to the number of iterations is important to mention that: (a) the bio-inspired algorithms used the maximum number of iterations (120) in each search and (b) the sequential algorithms never reached the maximum number of iterations (120). Therefore, the number of iterations is irrelevant to develop comparisons among bio-inspired algorithms; otherwise, the same is important to compare both sequential and bio-inspired algorithms. The search time in Figure 7 shows that: (a) solutions founded with error classification fitness function are more susceptible to long search times. The data have shown a relation of 250:1 between the search time of the classification error fitness function and the search time of the correlation factor fitness function. This was already expected due to the complexity associated with the supervised and non-supervised fitness functions (see fitness functions in Background Section); (b) the major and minor search time of the algorithms, regardless of the fitness function, are for SFFS and PSO respectively. The difference between these two algorithms is significant and indicates that the SFFS algorithm consumes almost four times more iterations than the PSO algorithm, for the case of maximum number of iterations = 120. The PSO algorithm is 14 percent better than the ABC algorithm, when correlation factor was used as fitness function. The difference (with respect to SFS) reaches 31 percent when classification error is used as fitness function.

This advantage of PSO over the other evaluated search algorithms is consequence of the low number of evaluations of the fitness function in each iteration of the PSO algorithm. The fitness values shown in Figure 8 indicate the following behaviors: (a) the fitness computed on the classification error fitness function was lower than that computed with correlation factor as fitness function; (b) the optimal solutions computed with sequential algorithms were closer to the global minimum than the ones computed with the bio-inspired algorithms. These results suggest a superiority of the solutions computed with sequential algorithms. In order to generalize that behavior, it is necessary to test the bio-inspired algorithms using other configuration parameters; (c) the fitness reached with the sequential algorithms SFS and SFFS are similar and, therefore, we can suggest that the nesting effect associated with the SFS algorithm is not significant in the considered fitness functions.

Figure 9 shows the comparison of the performances reached when the patterns were tuned with the different transformation matrices $W_{R}^{\text{ij}}$ (*i* = 1,2; *j* = 1,…,4). The results indicate the following aspects: (a) the classification rates are similar to the previous ones published in [5] and the classification error of the conventional myoelectric control architecture is superior to the classification error of the myoelectric control architecture with iPCA tuning; (b) there are similar levels of classification error when transformation matrices $W_{R}^{i∙}$ were used (i.e. transformation matrices computed with the *i* fitness function and each of the search algorithms); and (c) there are differences between classification errors when transformation matrix $W_{R}^{∙j}$ was used (i.e. transformation matrices computed with the *j* search algorithm and each of the fitness functions).

The response presented in (a) validates the superiority of the iPCA tuned architecture over the conventional myoelectric control architecture. Otherwise, the (b) response was not expected. Due to the superiority of fitness that was computed with sequential algorithms (see Figure 8), greater differences between the classification errors were expected. For instance, that classification errors associated to transformation matrices computed with sequential algorithms ( $W_{R}^{\text{ij}}$ with *j* = 1,2) were less than classification errors associated to transformation matrices computed with bio-inspired algorithms ( $W_{R}^{\text{ij}}$ with *j* = 3,4). This suggests that finding of the minimal value for the fitness function is not a sufficient condition to guarantee minimal classification errors during the evaluation of the myoelectric control system.

Finally, the (c) response was expected, basically by two reasons: (*i*) the superiority of classification error over correlation factor in determining the discriminant information in the selected subsets. (*ii*) The superior fitness of computed solutions using systems with classification error fitness function (see Figure 8).

The confusion matrices displayed in Additional file 1: Table S5, Additional file 2: Table S6, Additional file 3: Table S7 and Additional file 4: Table S8 provide a direct comparison between the optimization alternatives used. Additional file 1: Table S5 and Additional file 3: Table S7 compare classification accuracy when the transformation matrices $W_{R}^{2j}$ were used (i.e. the transformation matrices computed with the correlation factor fitness function and the sequential and bio-inspired algorithms). Additional file 2: Table S6 and Additional file 4: Table S8 compare classification accuracy when the transformation matrices $W_{R}^{1j}$ were used (i.e. the transformation matrices computed with the classification error fitness function and the sequential and bio-inspired algorithms).

In Additional file 1: Table S5 and Additional file 3: Table S7, the values in white (left columns) show processing with SFS algorithm and the values in gray (right columns) show the results with SFFS algorithm. In Additional file 2: Table S6 and Additional file 4: Table S8, the values in white (left columns) show processing with PSO algorithm and the values in gray (right columns) show the results with ABC algorithm.

The results along the main diagonal are correct classifications (accuracy) and those lying outside of the main diagonal are incorrect classifications. Empty cells correspond to an error of 0% and the accuracies were rounded to the nearest tenth of a percent. The values on these tables confirm two trends: (a) similar levels on classification error when transformation matrices $W_{R}^{i∙}$ were used and (b) differences between classification errors when transformation matrices $W_{R}^{∙j}$ were used.

## Conclusions

This paper has presented and evaluated the use of bio-inspired optimization algorithms in the iPCA stage of Myoelectric Control Systems for hand prosthesis. The influence of fitness function and searching algorithms on myoelectric control systems with iPCA tuning were investigated in terms of optimization performance (e.g. running time, number of iterations, mean search time), solution fitness and classification performance. The alternatives considered for fitness functions were the following: classification error, correlation factor, and for search algorithms: SFS, SFFS, PSO and ABC. The experimental results suggest superiority on classification performance when reduced iPCA matrices computed with classification error fitness function were used. The results have also shown the independence of classification performance with regard to the search algorithm. However, a practical advantage was found in using PSO algorithm during the optimal search. This advantage is related to the computational time of the process during the parameter configuration stage and was corroborated for a particular set of configuration parameters in the algorithm. Future studies will be carried out to investigate other configuration parameters in the PSO algorithm as well as the effects of the selected subset length *N*_1_ on the classification performance.

## Nomenclature

MCSs Myoelectric Control Systems

iPCA individual principal component analysis

PCA principal component analysis

ABC artificial bee colony

PSO particle swarm optimization

EMG electromyographic signals

SFS sequential forward selection

SFFS sequential floating forward selection

MLP multilayer perceptron

NNs neural networks

LDA linear discriminant analysis

ULDA uncorrelated linear discriminant analysis

GMMs Gaussian mixture models

HMMs hidden Markov models

SVMs support vector machines

TD Time Domain

MAV mean absolute value

MAVS mean absolute value slope

ZC zero crossings

SSC slope sign changes

WL wave length

AR autoregressive

STFT small time Fourier transform

WT Wavelet transform

WPT Wavelet packets transform

TDAR concatenated TD and AR features

*x* generic EMG signal

*y* vector of classes of movement

$\overset{ˆ}{y}$ vector of predicted classes of movement

${\overset{ˆ}{y}}_{\text{ij}}$ vector of predicted classes of movement computed with $W_{R}^{\text{ij}}$

*e*_*ij*_ classification error of the system configured with the $W_{R}^{\text{ij}}$ transformation matrix

*x*_*v*_ validation data set of EMG signals

*x*_*tr*_ training data set of EMG signals

*x*_*tst*_ test data set of EMG signals

*x*_*i*_ position vector of the *i* particle and position vector of the *i* food source in the PSO algorithm and ABC algorithm respectively

*v*_*i*_ velocity vector of the *i* particle and new position vector around *x*_*i*_ in the PSO algorithm and ABC algorithm respectively

$p_{{\text{best}}_{i}}$ best individual position for the *i* particle in the PSO algorithm

*gfbest* first best position explored so far in the PSO algorithm

*w* inertial weight in the PSO algorithm

*gsbest* second best position explored so far in the PSO algorithm

*r*_1_, *r*_2_ and *r*_3_ random numbers uniformly distributed in the range (0,1)

*c*_1_ cognitive parameter in the PSO algorithm

*c*_2_and *c*_3_ social parameters in the PSO algorithm

*p*_*i*_ probability factor of each employed bee in the ABC algorithm

*C*_*lim*_ limit of cycles to improve a solution in the ABC algorithm

*C*_*max*_ maximum number of iterations and maximum number of cycles in the PSO algorithm and ABC algorithm respectively

*ϕ*_*ij*_ random number in the range [-1,1] that controls the production of food source positions around *x*_*i*_ in the ABC algorithm

*s* iPCA projected pattern from *x*

*M* number of observations of the EMG signal

*O*^**′**^ vector of possible optimal set of channels

*O* vector of optimal set of channels

*O*_*ij*_ vector of optimal set of channels computed with the *i* fitness function and the *j* search algorithm

*ε* minimum value of fitness function

*C*_*x*_ cross correlation factor

*R*_*x*_ correlation coefficient matrix

*F*_*c*_ normalized correlation factor

*j* index for the search algorithm

*i* index for the fitness function

*C* number of classes of movement

*N* length of the pattern vector

*N*_1_ length of the reduced size vector

*S* the number of particles and bees used during optimal search with bio-inspired algorithms

*W* PCA transformation matrix

*W*_*c*_ PCA transformation matrix for the *c* movement class

*W*_*iPCA*_ iPCA transformation matrix

*W*_*R*_ reduced iPCA transformation matrix

$W_{R}^{\text{ij}}$ reduced iPCA transformation matrix computed with the *i* fitness function and the *j* search algorithm

## Competing interests

The authors declare that they have no competing interests.

## Authors’ contributions

GAC implemented the modifications on the Matlab libraries to include the bio-inspired algorithms and developed the experiments. GAC and CHL sorted the results and were responsible for the statistical analysis. GAC, CHL and PAB participated in the design of the study and contributed to the result discussion. CJM and AFR helped drafting and revising the manuscript. All authors read and approved the final manuscript.

## Supplementary Material

Additional file 1 **Table S5 Confusion matrix for the sequential algorithms and correlation factor fitness function**. Click here for file

Additional file 2 Table S6 Confusion matrix for the bio-inspired algorithms and correlation factor fitness function. Click here for file

Additional file 3 Table S7 Confusion matrix for the sequential algorithms and classification error fitness function. Click here for file

Additional file 4 Table S8 Confusion matrix for the bio-inspired algorithms and classification error fitness function. Click here for file
