# Supplementary material for: An experimental evaluation of the incidence of fitness-function/search-algorithm combinations on the classification performance of myoelectric control systems with iPCA tuning
Source: Biomed Eng Online. 2013 Dec 27;12:133. doi: 10.1186/1475-925X-12-133 (PMC3880009; doi:10.1186/1475-925X-12-133)
Supplement: Additional file 2 — Table S6 Confusion matrix for the bio-inspired algorithms and correlation factor fitness function. [file 1475-925X-12-133-S2.pdf]

Table S6 Confusion matrix for the bio-inspired algorithms and correlation factor fitness function

	W. Pron.		W. Sup.		W. Flex.		W. Ext.		H. Open		K. Grip		C. Grip		P. Grip		F. P. Grip		T. Grip		No Mov.	
**W. Pron.**	97	97	0.1	0.2	0.1	0.1		0.1	0.4	0.2	0.5	0.4	0.2	0.1	0.1	0.1	1	1			0.7	0.8
**W. Sup.**	0.1	0.1	98.2	98.2				0.1	0.1	0.1	0.1		0.7	0.8			0.1	0.1			0.6	0.5
**W. Flex.**	0.7	0.9	0.2	0.3	97.3	**97.4**	0.3	0.1	0.6	0.3	0.1	0.1	0.1	0.1	0.1			0.2	0.1	0.1	0.4	0.5
**W. Ext.**	0.1	0.5	0.1	0.1			**98.8**	98.3		0.1				0.1		0.1	0.4	0.3			0.6	0.4
**H. Open**	3.6	4	0.2	0.1			0.1	1	92	92		0.1	0.1				1.6	0.4	0.1	0.1	2.3	2.3
**K. Grip**											**94.6**	94.1	1.2	0.9	3	3.8	0.5	0.8	0.5	0.3	0.1	0.1
**C. Grip**	0.5	0.7									1.1	0.9	92.3	**93.4**	0.2	0.3	2.8	2.5			3.2	2.2
**P. Grip**											4.6	4.6	0.1	0.3	**94.9**	94.5	0.1	0.2	0.2	0.3		
**F. P. Grip**	0.3	0.2								0.1	1	0.6	3.6	4.3	0.8	0.8	**94.2**	94				0.1
**T. Grip**									0.8	1	0.2	0.4	0.2	0.4	0.2	0.4		0.1	**98.4**	97.6		
**No Mov.**	0.1	0.1	1.2	1.5			0.2	0.1	0.1	0.1			0.4	0.4			1.1	1.2	0.3	0.1	96.6	96.6

Shows the distribution of errors for the 10 channels, eleven class problem and intact limbed subjects. The values in white (left columns) show the percents reached with the PSO algorithm. The values in gray (right columns) show the percents reached with the ABC algorithm. Empty cells correspond to an error of 0%.
